# Serum IL-5 and IFN-*γ* Are Novel Predictive Biomarkers for Anti-PD-1 Treatment in NSCLC and GC Patients

**DOI:** 10.1155/2021/5526885

**Published:** 2021-06-18

**Authors:** Qiu Zhao, Yanzhi Bi, Huiting Sun, Min Xiao

**Affiliations:** ^1^Department of Oncology, Changzhou Tumor Hospital Affiliated to Soochow University, Changzhou 213000, China; ^2^Changzhou Second People's Hospital, Changzhou 213000, China

## Abstract

**Background:**

Because responses of patients with cancer to immunotherapy can vary in success, effective biomarkers are urgently needed for predicting clinical response with anti-PD-1 treatment. We aimed to evaluate the IL-5 and IFN-*γ* level with the response of anti-PD-1 blockade in non-small-cell lung cancer (NSCLC) and gastric cancer (GC).

**Methods:**

Metastatic NSCLC and GC patients treated with anti-PD-1 monoclonal antibody were studied. Blood samples were taken before PD-1 McAb treatment, after the first cycle treatment, and during efficacy evaluation. The association between IL-5 and IFN-*γ* levels and clinical response were analyzed by the nonparametric Wilcoxon matched-pairs ranked tests. The progression-free survival (PFS) time was obtained by imaging evaluation and telephone follow-up of all the patients. Kaplan-Meier and the log rank test were used to plot the survival curve.

**Results:**

IL-5 and IFN-*γ* levels were detected in the peripheral blood of 40 NSCLC and 35 GC patients who have received anti-PD-1 treatment. In effective group, IL-5 and IFN-*γ* levels at best response points significantly decreased (*P* < 0.001) compared with pretherapeutic levels in NSCLC and GC patients with lymph node or distant metastasis. Compared with pretherapeutic levels, IL-5 and IFN-*γ* levels largely increased as the tumor progresses (*P* < 0.01). Higher IL-5 and IFN-*γ* levels before treatment indicated shorter progression-free survival in patients with NSCLC metastasis (*P* = 0.007, *P* = 0.0111). Moreover, their levels also accurately reflected the pseudoprogression of two NSCLC patients to anti-PD-1 treatment.

**Conclusions:**

Our results suggested that serum IL-5 and IFN-*γ* levels could be an effective indicator for predicting clinical efficacy and survival with anti-PD-1 blockade in NSCLC and GC patients.

## 1. Introduction

Program Death 1(PD-1) expressed on T cells, B cells, and myeloid cells negatively regulates immune response. Deficiency of PD-1 leads to immune dysfunction and autoimmune disease [1]. Program Death Ligand 1(PD-L1), its primary ligand, belongs to B7 gene family and is variably expressed by lymphoid and nonlymphoid cells. The combination of PD-L1 and PD-1 leads to inhibition of T cell-mediated cytokine secretion and lymphocyte recruitment [2]. When PD-L1 is widely expressed on tumor cells, it will provide an effective inhibitory tumor microenvironment [3]. PD-1/PD-L1 blocking antibodies provide a promising treatment for various cancers [4]. However, approximately 70%-80% of patients have failed to respond to this therapy, and excellent efficacy happened only with limited tumor types [5]. In addition, due to delayed kinetics and atypical response models, approximately 15% of patients have experienced pseudoprogression, which means that when images show tumor enlargement, the patients are clinically stable or improved [6].

These abnormal response patterns make it difficult to distinguish the effective patients from noneffective patients in the early stages of treatment. Therefore, it is essential to identify more accurate biomarkers to predict the response to PD-1 McAb treatment. This can benefit patients who experience tumor shrinkage after the treatment.

Immune interferon (IFN), also known as IFN-*γ*, is an potent anticancer cytokine secreted from CTLs, NK cells, NKT cells, and *γδ* T cells [7, 8]. IFN-*γ* plays an important role in the immune responses to infection and cancer. It can play its immunomodulatory effect by improving MHC-mediated antigen presentation, reinforcing type 1 T helper cell (Th1) responses, regulating leukocyte trafficking, facilitating Toll-like receptor signaling, and enhancing the antitumor and antimicrobial functions [9].

IL-5 is mainly produced by T helper-2 (Th2) lymphocytes and group 2 innate lymphoid cells (ILC2). It can increase antibody secretion through promoting the differentiation and growth of B cells and enhance the humoral immune response mediated by Th2 cells. Immunity to tumors is mainly governed by Th1-mediated cellular immunity. If Th1-Th2 drift occurs, it will lead to produce an immunosuppressive and development of cancer [10].

In our study, we investigated the correlation between serum IFN-*γ* and IL-5 levels and the clinical response to anti-PD-1 mAbs in NSCLC and GC patients.

## 2. Methods

### 2.1. Patients and Response Criteria

From January 2017 to December 2019, a total of 75 patients with metastatic NSCLC and GC (57 males and 18 females) were enrolled in the Changzhou Tumor Hospital affiliated to Soochow University, including 40 patients with metastatic NSCLC and 35 patients with metastatic GC. The average age of the 75 patients was 65.5 years (range from 23 to 81 years). All patients gave informed consent before being enrolled in our study. Our study obtained ethical approval from the Ethics Committee of Changzhou Cancer Hospital. The experimental protocol reported in this paper is in accordance with World Medical Association Declaration of Helsinki on Ethical Principles for Medical Research Involving Human Subjects. Patients were enrolled with the following criteria: (1) clinical diagnosis of stage IV NSCLC or GC; (2) receive anti-PD-1 mAb treatment regardless of whether combinate with chemotherapy or molecular targeted therapy; (3) meet Eastern Cooperative Oncology Group (ECOG) physical status 0-2; and (4) good compliance and voluntary accept cytokine testing.

According to the RECIST 1.1 standard (11), the complete disappearance of the target lesions is evaluated as complete response (CR), a more than 30% reduction of total target lesions is evaluated as partial response (PR), a more than 20% increase of total target lesions is evaluated as progressive disease (PD), and a reduction less than 30% or an increase less than 20% in the sum of target lesions was assessed as stable disease (SD). Disease control rate (DCR) is equal to the ratio of CR+PR+SD cases to the total number of cases, and all of these have been maintained for more than 4 weeks. Two NSCLC patients were identified as pseudoprogression (after a first treatment cycle, a more than 20% increase of the tumor size that was assessed as a partial response by imaging system). Progression-free survival (PFS) is the time from the beginning of PD-1 monoclonal treatment to the first disease progression by imaging evaluation. The detailed clinical characteristics of the patients are described in Table 1.

### 2.2. Sample Collection

Blood samples were collected from each patient before PD-1 McAb treatment, after the first cycle treatment, and in each subsequent radiographic evaluation.

### 2.3. Flow Cytometry

Prepare 100 *μ*l of sample solution, including 25 *μ*l of assay buffer, 25 *μ*l of each sample, 25 *μ*l of premixed magnetic beads, and 25 *μ*l of detection antibody (R 701001, Qingdao Raisecare Biological Technology Co. Ltd, China). Then, shake on a plate shaker (about 500 rpm) for 2 hours at room temperature. After that, 25 *μ*l of SA-PE was added to each test tube and shaken (approximately 500 rpm) for 30 minutes at room temperature. 500 *μ*l of 1X wash buffer was added to all test tubes and centrifuge for 5 minutes at 300-500 g. Remove the supernatant. 150 *μ*l of 1X wash buffer was added to all test tubes. Vortex for 30 seconds to resuspend the beads. All operations need to be protected from light. Read the sample on the flow cytometer (Beckman Coulter Epics XL Cytometer).

### 2.4. Statistical Analyses

The statistical analysis was performed by GraphPad Prism 8.2.1 software (GraphPad Software Inc., USA). The association between IL-5 and IFN-*γ* levels and clinical response were analyzed by the nonparametric Wilcoxon matched-pairs ranked tests. The PFS data were obtained by standardized imaging assessment of all patients. The analysis of survival situation was followed up to January 31, 2020. Kaplan-Meier and the log rank test were used to plot the survival curve. *P* < 0.05 was considered as statistically significant difference.

## 3. Results

### 3.1. Patient Population

From January 2017 to December 2019, a total of 75 consecutive patients received anti-PD1 therapy at Changzhou Tumor Hospital affiliated to Soochow University and met study inclusion criteria. These patients diagnosed with NSCLC (40/75) or GC (35/75) took 200 mg anti-PD1 McAb every two or three weeks. Patients were followed up for at least 24 months. According to RECISTv1.1 standard, the DCR of patients diagnosed with NSCLC or GC was 72.5% and 48.5%, respectively. Two NSCLC patients were identified as pseudoprogression (after a first treatment cycle, a more than 20% increase of the tumor size which was assessed as a partial response by imaging system).

### 3.2. Serum IL-5 and IFN-*γ* Levels Associated with Response to Anti-PD-1 Treatment in Metastatic NSCLC Patients

**Figure 1:**
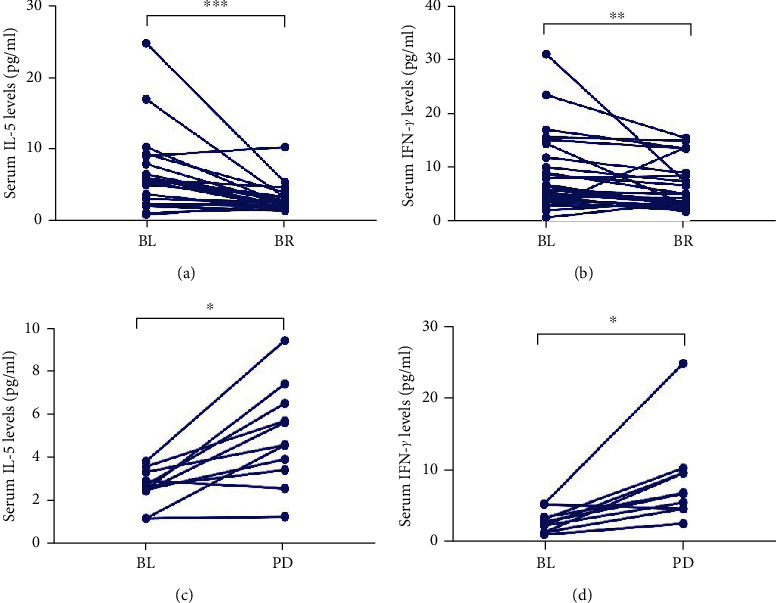
Serum IL-5 and IFN-*γ* levels reflect clinical response to anti-PD-1 mAbs in NSCLC patients. Serum IL-5 and IFN-*γ* levels were assessed at baseline (BL), best response (BR), and progressive disease (PD). (a) The median IL-5 levels at BR were lower than that at BL in the effective group; (b) the median IFN-*γ* levels at BR were lower than that at BL in the effective group; (c) the median IL-5 and IFN-*γ* levels at PD were higher than that at BL in the ineffective group; (d) the median IL-5 and IFN-*γ* levels at PD were higher than that at BL in the ineffective group. Statistical difference between median IL-5 and IFN-*γ* levels in different time-points was made by nonparametric Wilcoxon matched-pairs ranked tests. ^∗^*P* < 0.05; ^∗∗^*P* < 0.01; ^∗∗∗^*P* < 0.001.

Serum IL-5 and IFN-*γ* levels were tested in 40 patients with NSCLC during anti-PD-1 mAb treatment. In the effective group (CR+PR+SD) (*n* = 29), the median IL-5 level at best response (BR) was 2.56 pg/ml, which was significantly lower than before treatment (6.50 pg/ml) (*P* < 0.001, Figure 1(a)). Similarly, the median IFN-*γ* level at BR was 6.27 pg/ml, which was significantly lower than before treatment (8.74 pg/ml) (*P* < 0.001, Figure 1(b)). In the ineffective group (PD) (*n* = 11), the median IL-5 and IFN-*γ* levels increased significantly as the disease progressed (baseline 2.43 pg/ml (IL-5), 2.77 pg/ml (IFN-*γ*) versus PD 4.87 (IL-5), 8.24 pg/ml (IFN-*γ*), *P* < 0.05, *P* < 0.05, respectively) (Figures 1(c) and 1(d)).

### 3.3. Serum IL-5 and IFN-*γ* Levels Associated with Response to Anti-PD-1 Treatment in Metastatic GC Patients

**Figure 2:**
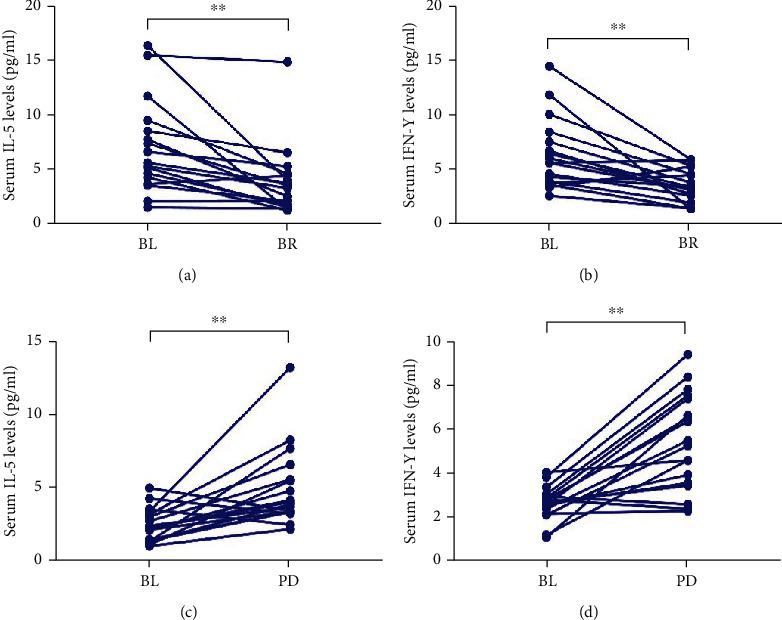
Serum IL-5 and IFN-*γ* levels reflect clinical response to anti-PD-1 mAbs in metastatic GC patients. Serum IL-5 and IFN-*γ* levels were assessed at BL, BR, and PD. (a) The median IL-5 levels at BR were lower than that at BL in the effective group; (b) the median IFN-*γ* levels at BR were lower than that at BL in the effective group; (c) the median IL-5 and IFN-*γ* levels at PD were higher than that at BL in the ineffective group; (d) the median IL-5 and IFN-*γ* levels at PD were higher than that at BL in the ineffective group. Statistical difference between median IL-5 and IFN-*γ* levels in different time-points was made by nonparametric Wilcoxon matched-pairs ranked tests. ^∗^*P* < 0.05, ^∗∗^*P* < 0.01, ^∗∗∗^*P* < 0.001.

We also detected IL-5 and IFN-*γ* levels in 35 patients with metastatic GC. In the effective group (CR+PR+SD) (*n* = 17), the median IL-5 level at BR was 3.78 pg/ml, which was significantly lower than before treatment (7.07 pg/ml) (*P* < 0.01, Figure 2(a)). Similarly, the median IFN-*γ* level at BR was 6.29 pg/ml, which was significantly lower than before treatment (3.27 pg/ml) (*P* < 0.01, Figure 2(b)). In the ineffective group (*n* = 18), the median IL-5 and IFN-*γ* levels increased significantly as the disease progressed (baseline: 2.37 pg/ml (IL-5), 2.81 pg/ml (IFN-*γ*) versus BR: 4.67 pg/ml (IL-5); 7.51 pg/ml (IFN-*γ*); *P* < 0.01, *P* < 0.01, respectively) (Figures 2(c) and 2(d)).

### 3.4. Predictive Value of Serum IL-5 and IFN-*γ* Levels for Pseudoprogression

**Figure 3:**
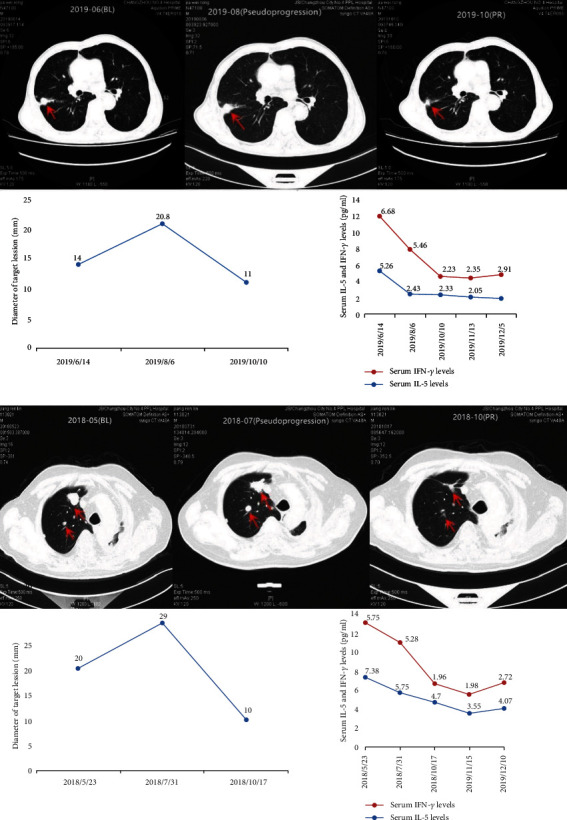
Serum IL-5 and IFN-*γ* levels reflect pseudoprogression in 2 metastatic NSCLC patients during anti-PD-1 mAb treatment. Serum IL-5 and IFN-*γ* levels were tested at baseline, at pseudoprogression diagnosis (first increase > 25% in tumor lesion size) and at subsequent imaging evaluations that fulfilled criteria of partial response (PR). Although imaging-evaluated increases in the lesion size during pseudoprogression, serum IL-5 and IFN-*γ* levels decreased and remained below the baseline levels.

Serum IL-5 and IFN-*γ* levels were detected in two patients with NSCLC who were diagnosed as pseudoprogression before treatment and at the time of a pseudoprogression diagnosis. Interestingly, although the imaging evaluation showed that the tumor lesions increased during pseudoprogression, serum IL-5 and IFN-*γ* levels inversely decreased and remained below the baseline levels for a long time (Figures 3(a) and 3(b)).

### 3.5. Decreased Serum IL-5 and IFN-*γ* Levels Correlated with Positive PFS in NSCLC Patients Treated with Anti-PD-1 mAbs

**Figure 4:**
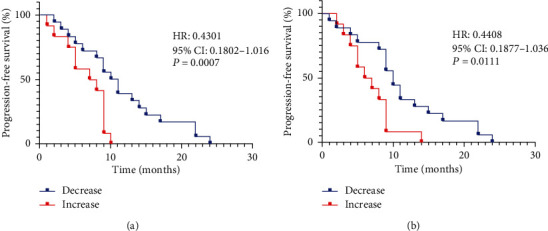
Serum IL-5 and IFN-*γ* levels associated with progression-free survival in metastatic NSCLC patients treated with anti-PD-1 mAbs. Patients were stratified into two subgroups (decrease and increase) based on IL-5 or IFN-*γ* levels at BL. Kaplan-Meier plots depict PFS differences between IL-5 (a) or IFN-*γ* (b) levels. Data were analyzed by the log-rank (Mantel-Cox) test.

Kaplan-Meier plots progression-free survival (PFS) stratified by early changes in the serum IL-5 and IFN-*γ* levels of NSCLC patients treated with anti-PD1 mAbs. It turned out that NSCLC patients whose serum IL-5 and IFN-*γ* initially decreased had longer progression-free survival than those who initially increased (median PFS (months): early decreases cohort: 11.2 (IL-5), 10.8 (IFN-*γ*) versus early increases cohort: 6.75 (IL-5), 7.3 (IFN-*γ*)) (Figures 4(a) and 4(b)). However, such PFS difference was not observed in metastatic GC cohort.

## 4. Discussion

Immunocheckpoint inhibitors mainly target the inhibitory immune response in the tumor microenvironment, and it takes a long time to experience tumor shrinkage or regression. Studies have shown that the response to anti-PD-1 mAbs usually took 12 weeks and the reaction time even longer in some cases [11]. In addition, immunocheckpoint inhibitor therapy can lead to atypical tumor responses such as pseudoprogression which may occur in up to 15% of cases. Pseudoprogression refers to an unusual radiologic imaging of tumor response where a more than 20% increase of target lesion size or the appearance of new lesions is not recognized as true disease progression [6]. It is crucial to identify biomarkers to predict the response of checkpoint blockades. The overexpression of PD-L1 on epithelial tumors is often treated with PD1/PD-L1 blocking antibodies. However, due to low prediction accuracy and dynamic changes, PD-L1 staining cannot be used to screen out patients sensitive to anti-PD-1 mAbs [12]. Tumor-infiltrating immune cells in the tumor microenvironment, gene analysis such as microsatellite instability, mismatch-repair deficiency, and tumor mutation burden may be important in predicting clinical benefits of PD-1/PD-L1 checkpoint blockades [13].

Peripheral blood collection is a less invasive, safe, convenient, and repeatable method for sample collection. Furthermore, peripheral blood can provide a systematic view of the host's immune status. In recent years, some biomarkers in the blood circulation have shown value in predicting the response of patients to immunotherapy, such as PD-1^+^CD8^+^ T cells [14], CD4^+^ T cells [15], TCR repertoire [16, 17], cell-free DNA, circulating tumor cells, and cytokines [18].

In this study, we demonstrated that serum IL-5 and IFN-*γ* levels were correlated with the efficacy of anti-PD-1 treatment in metastatic GC and NSCLC patients. Moreover, we elucidated that early changes in serum IL-5 and IFN-*γ* levels can predict the PFS in NSCLC patients treated with anti-PD-1 mAbs.

IL-5 and IFN-*γ* levels also can be used to distinguish pseudoprogression from true disease progression. It appeared that lymphocyte infiltration is associated with tumor-related lesions when pseudoprogression occurs. It is hard to identify subcellular changers of tumor lesions by imaging technology. In our study, we for the first time found that serum IL-5 and IFN-*γ* levels can be useful biomarkers reflecting the changes in tumor areas. Moreover, we introduced two cases of pseudoprogression. They initially showed an increase in tumor lesion, and a relatively significant reduction was evaluated by imaging techniques. When tumor lesions increased evaluated by imaging methods, serum IL-5 and IFN-*γ* levels were lower than baseline level. These results suggested that serum IL-5 and IFN-*γ* levels might become useful biomarkers to identify pseudoprogression. However, more pseudoprogresssion samples are needed to confirm this finding.

IL-5 is mainly produced by leukocytes including T cells, eoinophils, basophils, and natural helper cells [19]. Studies have found that innate IL-5-producing cells localized most abundantly in the lung and contributed to maintaining enough lung eosinophils. Given that eosinophils have proven to have antitumor activity, Ikutani et al. showed that innate IL-5-producing cells were increased in response to tumor invasion, and their regulation of eosinophils is critical to suppress tumor metastasis. Conversely, exogenous IL-5 treatment resulted in suppressed tumor metastasis and augmented eosinophil infiltration [10]. These newly identified innate IL-5-producing cells thus play a role in tumor surveillance through lung eosinophils and may contribute to development of novel immunotherapies for cancer. We found a close correlation between serum IL-5 levels and clinical efficacy of anti-PD-1 mAb treatment. This can be explained by the fact that when anti-PD-1 mAb treatment was effective, tumor burden and invasion range were subsequently reduced. Then, IL-5-producing cells are decreased by negative feedback, resulting in a decrease in IL-5 levels. This may also apply to the correlation between IFN-*γ* level and clinical response to anti-PD-1 mAb treatment. Moreover, potential inflammatory conditions should be considered in our study. In this regard, we have detected inflammatory factors including IL-1, IL-6, and TNF-*α* to help identify whether the increase of IL-5 and IFN-*γ* levels was caused by tumor progression or the concurrent inflammatory conditions. Results showed that none of these common inflammatory factors was associated with the clinical response to anti-PD-1 mAb treatment.

There was no correlation between pretreatment of IL-5 and IFN-*γ* levels and the efficacy of anti-PD-1 mAbs. It may be because the therapeutic responses to anti-PD-1 mAb treatment are complicated and involve multiple immune processes [20, 21]. In addition, the therapeutic response to anti-PD-1 mAbs has been suggested to be a critical state transition process of a complex system, which is hard to predict long-term in advance [22].

## 5. Conclusion

We found that serum IL-5 and IFN-*γ* levels were closely associated with tumor burden in metastatic NSCLC and GC patients receiving anti-PD-1 mAbs, so their expression levels can assess the efficacy of anti-PD-1 mAb treatment. Moreover, early changes of their levels correlated with progression-free survival in patients with metastatic NSCLC patients. Finally, IL-5 and IFN-*γ* levels had a better predictive value than imaging techniques in a small number of patients diagnosed with pseudoprogression. Metastatic NSCLC patients with initially decreased IL-5 and IFN-*γ* levels obtained positively clinical outcome and long PFS with anti-PD-1therapy. Our study provides effective biomarkers for the evaluation of efficacy and prognosis of anti-PD-1 mAb treatment; however, the number of patients in our study is limited, and further prospective validation including multiple types of cancers is required.

## Figures and Tables

**Table 1:** The clinicopathological parameters of patients.

Parameters	NSCLC	Gastric cancer
Gender, *n* (%)		
Male	34 (85)	23 (65.7)
Female	6 (15)	12 (34.3)
Age (year)		
⩽60	13 (32.5)	10 (28.5)
>60	27 (67.5)	25 (71.5)
Has smoking history	32 (80)	21 (60)
Brain metastasis	2	0
Eastern Cooperative Oncology Group status (ECOG), *n* (%)		
0	7 (17.5)	2 (5.7)
1	30 (75)	26 (74.2)
2	3 (7.5)	7 (20.1)
Adenocarcinoma	26 (65)	35 (100)
Squamous cell carcinoma	14 (35)	0
First-line treatment	18 (45)	9 (25.7)
Second- and third-line treatment	22 (55)	26 (74.2)
Complete remission (CR)	4 (10)	2 (5.7)
Partial remission (PR)	12 (30)	6 (17.1)
Stable disease (SD)	13(32.5)	9 (25.7)
Disease progression (PD)	9 (22.5)	18 (51.4)

## Data Availability

The data used in the study are available from the corresponding author upon request.
